# Impact of Plant Peptides on Symbiotic Nodule Development and Functioning

**DOI:** 10.3389/fpls.2018.01026

**Published:** 2018-07-17

**Authors:** Attila Kereszt, Peter Mergaert, Jesús Montiel, Gabriella Endre, Éva Kondorosi

**Affiliations:** ^1^Institute of Plant Biology, Biological Research Centre, Hungarian Academy of Sciences, Szeged, Hungary; ^2^Institute of Integrative Biology of the Cell, UMR 9198, CNRS – CEA – Université Paris-Sud, Gif-sur-Yvette, France

**Keywords:** legume-rhizobium symbiosis, nodule development, signaling peptides, NCR, CLE, CEP, GRP

## Abstract

Ribosomally synthesized peptides have wide ranges of functions in plants being, for example, signal molecules, transporters, alkaloids, or antimicrobial agents. Legumes are an unprecedented rich source of peptides, which are used to control the symbiosis of these plants with the nitrogen-fixing *Rhizobium* bacteria. Here, we discuss the function and the evolution of these peptides playing an important role in the formation or functioning of the symbiotic organs, the root nodules. We distinguish peptides that can be either cell-autonomous or secreted short-range or long-range signals, carrying messages in or between plant cells or that can act as effectors interacting with the symbiotic bacteria. Peptides are further classified according to the stage of the symbiotic process where they act. Several peptide classes, including RALF, DLV, ENOD40, and others, control *Rhizobium* infection and the initiation of cell divisions and the formation of nodule primordia. CLE and CEP peptides are implicated in systemic and local control of nodule initiation during autoregulation of nodulation and in response to the nutritional demands of the plant. Still other peptides act at later stages of the symbiosis. The PSK peptide is thought to be involved in the suppression of immunity in nodules and the nodule-specific cysteine-rich, GRP, and SNARP (LEED..PEED) peptide families are essential in the functioning of the nitrogen fixing root nodules. The NCRs and possibly also the GRP and SNARPs are targeted to the endosymbionts and play essential roles in the terminal differentiation of these bacteria.

## Introduction

**FIGURE 1:**
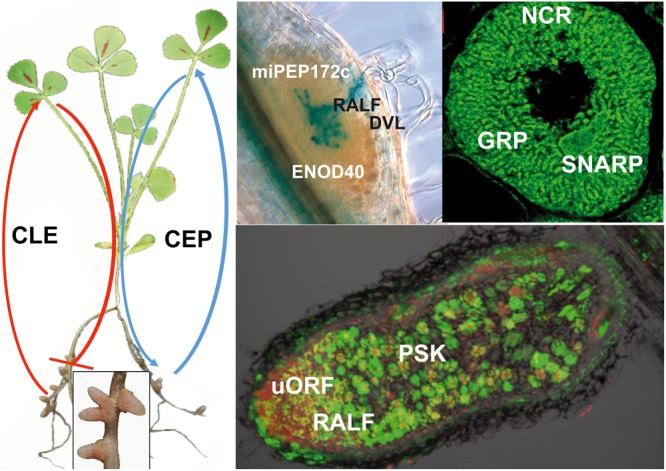
Peptides contributing to nodule formation. The left part of the figure presents a nodulated *M. truncatula* plant. The inset shows an enlarged image of nodules. The upper pictures show a section of a nodule primordium showing a network of infection threads stained in blue **(Left)**, and a symbiotic nodule cell densely packed with differentiated bacteroids **(Right)**. The lower image is a section of a mature nodule showing the rhizobia in green. The red staining shows plant cell nuclei, highlighting primarily the nodule meristem. Peptides involved in nodulation are indicated at their presumed site of action. CLE peptides are produced in response to already initiated nodules or by high nitrate. They are systemic signals received in the shoot by the HAR1/SUNN/NARK receptor-like kinases, which in return produce a shoot-derived signal that inhibits further nodulation in the roots. CEP peptides on the other hand are produced in the roots and their production is enhanced by low nitrogen. The peptides are perceived by the CRA2 receptor-like kinase and stimulate nodulation through a systemic mechanism. The RALF and DLV peptides negatively affect the infection process in early stages of nodule development. During later stages, RALF has a negative, while PSK has positive effect on the process. ENOD40 and miPEP172c affect nodule primordium formation. uORFp1 participates in the control of the meristem maintenance. NCR peptides and potentially also the GRP and SNARP peptides control bacteroid differentiation and functioning.

Ribosomally synthesized peptides with biological functions are arbitrarily (and loosely) defined as gene-encoded small proteins of 2 to about 100 amino acids. Research on peptide-mediated signaling processes and other peptide functions in plants has gained momentum in the last decade [for a review, see Tavormina et al. (2015)]. Crucial importance of peptides has been demonstrated in embryogenesis (Costa et al., 2014), fertilization (Okuda et al., 2009; Higashiyama, 2010; Mecchia et al., 2017; Ge et al., 2017), cell expansion (Haruta et al., 2014; Murphy and De Smet, 2014), cell differentiation (Butenko et al., 2003; Hunt et al., 2009; Matsuzaki et al., 2010; Sugano et al., 2010; Lee et al., 2015; Santiago et al., 2016; Doblas et al., 2017; Nakayama et al., 2017), immunity (Constabel et al., 1995; Pearce et al., 2001; Huffaker et al., 2006; Hou et al., 2014; Stegmann et al., 2017), nutrition (Tabata et al., 2014; Ohkubo et al., 2017), as well as in other processes (Whitford et al., 2012; Takahashi et al., 2018). The field of *Rhizobium*-legume symbiosis is not lagging behind when it comes to discoveries of peptides with key roles in the nodulation process (Djordjevic et al., 2015). In this review, we summarize these peptide signals and peptide effectors and the present knowledge on their identified or predicted functions in symbiosis (**Figure 1**).

In response to nitrogen starvation, plants from the Leguminosae family can establish symbiosis with their *Rhizobium* partners resulting in the development of root nodules and within the nodule plant cells, the conversion of the bacteria into nitrogen fixing bacteroids. For the initiation of the symbiosis and finding the appropriate *Rhizobium* bacterium in the soil, legume plants excrete from their roots flavonoids and isoflavonoids acting as inducers of nodulation genes in their symbiont. Activation of nodulation genes leads to the production of bacterial signal molecules, the Nod factors, which induce nodule organogenesis in the host plant and are required for the infection process as well. Already at this stage, several plant peptides affect the plant susceptibility to infection and nodulation and participate in the regulation of these first developmental and differentiation steps. Moreover, the plant invests only in the number of nodules required to satisfy its nitrogen needs. The nodule numbers are negatively controlled by autoregulation of nodulation and by high nitrate using CLV3/ESR-related (CLE) peptides exerting a systemic negative regulation on nodulation *via* root- and shoot-derived signaling. Members of the C-terminally encoded peptide (CEP) family, on the contrary, exert a positive effect on nodulation in response to low nitrogen availability.

While these peptide signals have general and conserved roles and some of their members were recruited to serve in the nitrogen fixing symbiosis as well, other large secreted symbiotic peptide families, which evolved specifically in certain legume lineages, are linked to irreversible, or terminal, differentiation of the bacteroids. These bacteroids are not able to switch from the symbiotic state to free living one and are thus non-cultivable. They are also morphologically different from the free living cells, often exhibiting remarkable cell growth and change in cell shape, having an amplified genome and altered cell envelope with increased membrane permeability. Such terminal bacteroid differentiation occurs in several but not all branches of the Leguminosae family and, so far, has only been studied in legumes of the Inverted Repeat Lacking Clade (IRLC) from the Papilionoideae subfamily and in the phylogenetically distant *Aeschynomene* genus of Dalbergoid legumes. In these legumes, these peptides direct terminal bacteroid differentiation, which is indispensable for nitrogen fixation. In other legumes, where the fate of the bacteroids is reversible and endosymbionts can return to the free-living life, there is no change in morphology, size, DNA content, and membrane properties of the bacteria, and these legumes lack genes coding for the abovementioned symbiotic peptide families in their genome. The large majority of these peptides are nodule specific cysteine-rich (NCR) peptides, but there are also glycine-rich peptides (GRPs) as well as small nodulin acidic RNA-binding peptides (SNARPs or also named LEED..PEED according to a conserved amino acid motif).

## Plant Peptides Involved in Infection and Nodule Organogenesis

### Regulators Encoded by Peptide-Coding Genes

#### Rapid Alkanization Factor (RALF) Family

The *Medicago truncatula MtRALFL1* gene was identified in a transcriptome screen for early Nod factor-induced genes using a double supernodulating mutant line (Combier et al., 2008b). The gene encodes a member of the RALF cysteine-rich and secreted peptide family, which comprises 15 members in *M. truncatula* (Silverstein et al., 2007; de Bang et al., 2017). Peptides of the RALF family are known in plants to control immunity as well as cell expansion, notably in root hair and pollen tube growth (Murphy and De Smet, 2014; Haruta et al., 2014; Ge et al., 2017; Mecchia et al., 2017; Stegmann et al., 2017). The secretion of RALF peptides indicates that they function in cell-to-cell signaling what they indeed do through the interaction with cell membrane located receptors like the receptor-like kinase FERONIA. Notably, FERONIA and other RALF-binding receptors are related receptor-like kinases that have an ectodomain (ligand-binding extracellular domain of the receptor) composed of two malectin-like domains (Li et al., 2016). The RALF peptides are bound by the malectin-containing ectodomains but the molecular details of the interaction need to be further clarified.

The *MtRALFL1* gene is induced by Nod factor treatment of *M. truncatula* roots although this induction was only observed in the particular genetic background of the supernodulating *sunn-2 sickle* double mutant (Combier et al., 2008b). This *M. truncatula* line has a higher sensitivity to Nod factors and, therefore, allowed to detect responses that are not visible in a wild-type background. The involvement of the MtRALFL1 peptide in nodulation was further supported by overexpression of *MtRALFL1* in *M. truncatula* transgenic roots, which resulted in a drastic reduction of nodule number and an abnormally high number of aborted infection threads. Moreover, the few nodules initiated on the transgenic roots did not develop into mature, nitrogen fixing organs. Thus, MtRALFL1 controls infection thread formation and possibly other stages of nodule development (Combier et al., 2008b). Interestingly, infection threads have a polar growth mode similar to root hairs and pollen tubes whose growth is also affected by RALF peptides in other plant species. Furthermore, the nodulation receptor-like kinase NORK [also known as “doesn’t make infections 2” (DMI2) or “symbiosis receptor-like kinase” (SymRK)], which is part of the Nod factor receptor complex regulating infection thread formation, contains in its ectodomain a malectin-like domain (Endre et al., 2002; Antolín-Llovera et al., 2014). Although the overall ectodomain structure of NORK differs from the ectodomain of FERONIA and related receptor-like kinases by the presence of an additional leucine-rich repeat domain, it is tempting to speculate that the MtRALFL1 peptide targets the Nod factor receptor complex.

#### Medicago DEVIL (MtDVL1) Non-secretory Peptide

Another characterized regulatory peptide in *M. truncatula* is called MtDVL1. The *MtDVL1* gene was identified in the same transcriptome screen as the above described *MtRALFL1* gene (Combier et al., 2008b). MtDVL1 is homologous to the family of ROTUNDIFOLIA FOUR (ROT4) or DEVIL (DVL) peptides in Arabidopsis, which are conserved in plants (Guo et al., 2015). These peptides are non-secretory and are thought to function cell-autonomously (Ikeuchi et al., 2011). Understanding the biological function and mode of action of this family of peptides is limited. Loss-of-function or knock-down mutants show no noticeable phenotypes, likely due to gene redundancy in the family, but overexpression of several members of the family in Arabidopsis produces phenotypes suggesting the implication of DVL peptides in plant development (Narita et al., 2004; Wen et al., 2004; Ikeuchi et al., 2011; Valdivia et al., 2012; Guo et al., 2015). The function of the *MtDVL1* gene in symbiosis was also assessed by overexpression studies in transgenic *M. truncatula* roots, which led to a strong increase in the number of abortive infections in the root cortex and in line with this, a significant reduction of nodule formation suggesting that the MtDVL1 peptide has a negative regulatory role in nodulation and particularly in rhizobial infection (Combier et al., 2008b).

#### Phytosulfokine (PSK) Peptides

Phytosulfokines are five-amino acid peptides containing two sulfated tyrosine residues. These peptides are produced as preproproteins, which are secreted as sulfated precursors in the cell apoplast where they are further processed to the PSK peptide by a subtilisin serine protease (Srivastava et al., 2008; Komori et al., 2009). The PSK peptides are recognized by a receptor-like kinase and act primarily as growth-promoting factors (Matsubayashi et al., 2002) but also participate in the immune response of plants by either attenuating pattern-triggered immunity against biotrophs or promoting immunity against necrotrophic pathogens (Igarashi et al., 2012; Zhang et al., 2018). In *Lotus japonicus*, five *PSK* genes have been identified, two of which showed a nodule-specific expression found mainly in the rhizobium-infected symbiotic cells (Wang et al., 2015). Overexpression of one of the *PSK* genes in transgenic *L. japonicus* roots but also the external application of the PSK-α peptide to roots enhanced nodulation. *PSK* overexpression did not increase infection events or the number of initiated nodule primordia and, therefore, the enhanced nodulation was attributed to a stimulation of nodule growth from primordia by PSK. Moreover, PSK overexpression resulted in the downregulation of the jasmonate signal transduction pathway. Thus, the nodule-specific PSK peptides might be also important in nodules to suppress host defense responses against the rhizobia (Wang et al., 2015).

### Short Peptides Encoded by sORFs (sPEPs)

The peptides described so far are derived from genes whose major open reading frame (ORF) encodes the peptide or a peptide precursor. Recently, an additional source of peptides was recognized in short ORFs (sORFs) located in RNA molecules, which have another primary function (Andrews and Rothnagel, 2014; Hellens et al., 2016; Makarewich and Olson, 2017). These RNAs can be transcripts previously annotated as long non-coding RNAs (lncRNAs), primary microRNA transcripts (pri-miRNAs), or protein-encoding mRNAs. The translation of some of these sORFs has been demonstrated experimentally by translational GUS fusions, ribosome profiling, overexpression or mutational analysis of the transcript *in planta*, or by the immunological detection of the peptides. The biological activity of some sPEPs has been demonstrated by *in planta* application or *in vitro* biochemical activities. Examples of sORFs/sPEPs have been identified as regulators of nodulation.

#### Peptides Encoded by sORFs in pri-miRNAs (miPEPs)

A class of newly identified peptide regulators, discovered thus far in plants only, is encoded by microRNA (miRNA) genes. The miRNAs are 21–24 nt regulatory RNA molecules and function *via* base-pairing with complementary sequences in target mRNAs, mediating cleavage or inhibition of translation of the target. The miRNAs are transcribed as large pri-miRNAs, which are then processed into mature miRNAs. Based on case studies in Arabidopsis and *M. truncatula*, certain pri-miRNAs were reported to contain in their 5′ part functional sORFs encoding the so-called miPEPs (Lauressergues et al., 2015). Evidence for the production of the Arabidopsis miPEP165a and the *M. truncatula* miPEP171b was obtained by translational fusions with GUS and by western blots and immunolocalization with specific antibodies produced against the corresponding synthetic peptides. It was further shown by overexpression of the corresponding sORFs or external application of synthetic peptides to plants that miPEPs enhance specifically the transcription of their cognate primary transcript. They thereby form a positive feedback loop and increase the level of the corresponding miRNA, amplify the original effect, and reduce even more the expression of the miRNA target genes. Although miPEPs have been experimentally characterized only in a few cases, a survey of the sequences of plant pri-miRNAs indicates that they generally contain sORFs suggesting that miPEPs are commonly encoded by pri-miRNAs.

A miPEP was recently shown to control nodulation in soybean (Couzigou et al., 2016). Several miRNAs are known to regulate different stages of the nodulation process [reviewed in Couzigou and Combier (2016)]. One of these miRNAs is the soybean miR172c, which targets *NNC1* gene coding for an APETALA 2 transcription factor. The NNC1 transcription factor, which regulates the nodule-specific gene *ENOD40* (see below), negatively affects nodulation, and thus the miR172c expression stimulates nodulation by reducing NNC1 activity (Wang et al., 2014). Similarly as the earlier characterized pri-miRNAs of *M. truncatula* and Arabidopsis, the primary transcript of soybean miR172c encodes the miPEP172c. Intriguingly, it was found that watering soybean plants with a solution containing synthetic miPEP172c peptide resulted in an increase of nodule numbers. This enhanced nodulation correlated with a higher expression of the miR172c primary transcript and several marker genes of nodulation while the *NNC1* gene expression was significantly reduced (Couzigou et al., 2016).

#### Peptides Encoded by Upstream ORFs (uORFs)

Regulatory sPEPs can also be encoded by sORFs located within 5′ leader sequences of protein encoding mRNAs. These sORFs are commonly referred to as uORFs. uORFs are ubiquitous and have been identified in most eukaryotes. Close to 50% of mRNAs contain uORFs (Andrews and Rothnagel, 2014; Hellens et al., 2016). Ribosome profiling and proteomics in mammalian and human cells revealed that many of the uORFs are indeed translated but only few have been functionally analyzed. A common function of these characterized uORFs is to attenuate the translation of their associated downstream coding ORF by stalling of the ribosomes on the 5′ leader sequence (Andrews and Rothnagel, 2014).

Such a uORF and its encoded sPEP have an essential role in the nodule meristem maintenance in *M. truncatula*. The NF-YA1 transcription factor in *M. truncatula* (also known as MtHAP2-1) controls nodule meristem function (Combier et al., 2006). The *NF-YA1* gene is expressed in the meristem and its spatial expression profile is finely regulated by two mechanisms that repress expression of *NF-YA1* in the adjacent infection zone of the nodule (zone II). In younger nodules, the *NF-YA1* gene is negatively regulated in zone II by the miR169 miRNA (Combier et al., 2006). In older nodules, a second mechanism takes over the negative regulation in zone II. An alternatively spliced mRNA of the *NF-YA1* gene becomes expressed in this zone. The alternative splicing of the first intron of the *NF-YA1* gene results in a transcript with a long 5′ leader sequence containing an uORF encoding the 62 amino acids peptide called uORF1p (Combier et al., 2008a). The translation of this peptide was supported by translational fusions of uORF1p with GUS. The functionality of the uORF1p peptide was investigated with its overexpression, which reduced the expression of the *NF-YA1* gene resulting in the development of aberrant nodules lacking tissue differentiation. Moreover, specific binding of the uORF1p peptide was demonstrated *in vitro* to the 5′ region of the *NF-YA1* transcript. Together, the data indicate that the synthesis of the uORFp1 peptide in zone II reduces in *trans* the mRNA levels of its cognate *NF-YA1* gene and thus in combination with the miR169, it restricts the expression of the gene to the nodule meristem. Interestingly, this in-*trans-*mode-of-action of the uORFp1 peptide on mRNA levels is unique because other described uORFs are *cis*-acting and inhibit translation of the downstream ORF by ribosome stalling (Andrews and Rothnagel, 2014).

#### Short Peptide of Early Nodulin ENOD40

sORFs are also present in RNAs annotated as lncRNAs, which are transcripts that do not encode a longer protein. In most cases, it is difficult to predict the significance of the sORFs in these lncRNAs (Andrews and Rothnagel, 2014). Nevertheless, a few examples of sPEPs encoded by these transcripts have been reported in plants (Tavormina et al., 2015). One of these sPEP and lncRNA encoding plant genes is the well-studied but still enigmatic *ENOD40* gene of legumes. *ENOD40* expression is induced in the incipient nodule primordium, and its expression is under the control of the early symbiosis signaling cascade (Frugier et al., 2008). Both *M. truncatula* and *L. japonicus* have two *ENOD40* gene copies and their downregulation or overexpression in transgenic plants result in a reduced or enhanced initiation of nodule primordia, respectively (Charon et al., 1999; Kumagai et al., 2006; Wan et al., 2007). Thus, the genes play a key role in the initiation of nodules and the establishment of the nodule primordium. The *ENOD40* gene lacks a long ORF but several sORFs are present, some of which are conserved among plant species, notably two sORFs encoding the peptides ENOD40-I (13 amino acids) and ENOD40-II (27 amino acids) (Sousa et al., 2001). The translatability of the sORFs was suggested by *in vivo* translational GUS fusions expressed in *Medicago sativa* roots. Moreover, a soybean ENOD40 peptide could be detected by western blotting (Röhrig et al., 2002). Ballistic microtargeting of the *ENOD40* gene into cortical root cells of *M. sativa* was found to induce cell divisions, which correspond to the early steps of nodule initiation (Charon et al., 1997; Sousa et al., 2001). *ENOD40* variants were used in this assay to demonstrate that both the ENOD40-I and ENOD40-II peptides as well as a structured RNA region of the transcript are involved in the elicitation of cortical cell divisions (Sousa et al., 2001). Another reported cellular activity of the *M. truncatula ENOD40* gene is the re-localization of the RNA-binding protein MtRBP1 from the nucleus into the cytoplasm (Campalans et al., 2004). The re-localization of MtRBP1 is dependent on the *ENOD40* RNA. However, the *ENOD40*-encoded peptides do not seem to be involved in this activity because mutant *ENOD40* genes where the translational initiation ATG codons of the peptides were mutated, were still able to induce the cytoplasmic localization of MtRBP1. Moreover, Laporte et al. (2010) reported binding of the *ENOD40* RNA molecule also with the SNARP peptides, which are further described in detail below. One can speculate that forming *ENOD40* RNA–protein interactions may be related to the facilitation of the translation of small proteins like the SNARPs. The picture is even more complex, as it was also found that the soybean ENOD40 sPEPs, named ENOD40-A and ENOD40-B, covalently bind to sucrose synthase, thereby stimulating its sucrose cleavage activity and protein stability. This suggests that the ENOD40 peptides are involved in the control of sucrose use in incipient nodules (Röhrig et al., 2002, 2004). Because the activity of sucrose synthase in plant tissues and organs correlates with the sink strength of these tissues, the ability to attract sucrose, and because sucrose synthase is involved in nodule initiation and essential for effective nitrogen fixation in nodules, it was suggested that the ENOD40 peptides may increase the carbon sink strength in pre-dividing root cortical cells and in mature nodule tissues (Röhrig et al., 2002, 2004).

Thus altogether, it seems that the *ENOD40* genes of legumes act both as a structured RNA molecule and by encoding sPEPs (Bardou et al., 2011). However, how the reported activities of the RNA molecule or the sPEPs are related to the key *ENOD40* functions in the activation of cell divisions in the root cortex and nodule primordium initiation remains a conundrum that requires more investigations.

## Plant Peptides Regulating Nodule Number

### CLE Peptides, Signals in the Autoregulation of Nodulation

The plant has to maintain the balance between the gains and costs of the formation and functioning of the symbiotic nodule, which are energy and carbon demanding processes. That is why legumes control the number of nodules formed on their roots. Both available nitrogen (mainly nitrate) sources and newly forming nodules restrict the initiation and progression of nodule development in the zone susceptible for rhizobial infection (Pierce and Bauer, 1983). Kosslak and Bohlool (1984) demonstrated with split-root inoculation experiments the existence of a long-distance signaling mechanism called autoregulation of nodulation (AON), which prevents the formation of new nodules on the whole root system after the initiation of nodule development at the first inoculation site [for review, see Reid et al. (2011) and Mortier et al. (2012)]. Using nodule excision experiments (Nutman, 1952; Caetano-Anolles and Gresshoff, 1990) and various split-root approaches including the use of bacterial and plant mutants as well as Nod factors revealed that this systemic regulatory signal is generated very rapidly after root hair curling and before the initiation of visible cortical and pericycle cell divisions, while its strength increases with progression of development (Kosslak and Bohlool, 1984; Mathews et al., 1989; Olsson et al., 1989; Caetano-Anolles and Gresshoff, 1990, 1991; van Brussel et al., 2002; Li et al., 2009). After the isolation of plant mutants defective in nitrate- and autoregulation of nodulation (Carroll et al., 1985; Park and Buttery, 1989; Wopereis et al., 2000; Penmetsa et al., 2003), it was demonstrated with the help of grafting experiments (Delves et al., 1986) that the shoot plays an important role in AON. It was proposed that in the developing nodule, the graft-transmissible signal cue (Q) is produced and is transported to the shoot where it induces the synthesis of the shoot-derived inhibitor (SDI), which suppresses nodulation (Delves et al., 1986; Caetano-Anolles and Gresshoff, 1991). In addition, the nitrate-tolerant phenotype of the mutants indicated that nitrate- and autoregulation pathways share genetic components. Map-based cloning of the mutated genes (*HAR1* in *L. japonicus, NARK* in *Glycine max, SUNN* in *M. truncatula*) identified a receptor kinase (Nishimura et al., 2002; Searle et al., 2003; Schnabel et al., 2005) that is the closest legume homolog of the Arabidopsis Clavata1 (CLV1) receptor (Clark et al., 1997). Also by genetic analysis, the receptor-like kinases KLAVIER and the membrane protein CLV2 were later identified as likely co-receptors of HAR1 in *L. japonicus* (Miyazawa et al., 2010; Krusell et al., 2011). Arabidopsis CLV1 functions in a protein complex controlling stem cell proliferation by short-distance signaling in shoot apices. As CLV1 is known to act as a receptor for the shoot apical meristem regulator CLV3 of the CLV3/ESR-related (CLE) small secreted peptide family (Fletcher et al., 1999; Cock and McCormick, 2001), it was immediately hypothesized that the ligand of HAR1, the signal Q, might be a CLE peptide (Nishimura et al., 2002). Indeed, it was shown that a few genes from the gene family encoding CLE peptides in legumes have Nod factor-dependent nodule-enhanced or even -specific and/or nitrate-dependent expression, and their ectopic expression led to systemic HAR1/SUNN/NARK-mediated repression of nodulation by interference with the nodulation signaling pathway (Okamoto et al., 2009; Mortier et al., 2010; Reid et al., 2011; Saur et al., 2011). Interestingly, the *L. japonicus CLE-RS2* gene plays a role in both nitrate- and nodulation dependent regulation, and its expression is under dual regulation by the Nod factor signal transduction pathway during nodulation *via* the NIN transcription factor and independently from it by nitrate exposition *via* the NIN-like transcription factor NRSYM1 (Okamoto et al., 2009; Soyano et al., 2014; Nishida et al., 2018). In contrast, soybean responds to nodulation by the expression of the *GmRIC1* and *GmRIC2* genes, while nitrate induces the expression of *GmNIC1*, another CLE encoding gene (Reid et al., 2011). The translated products of the *CLE* genes undergo extensive post-translational modifications and proteolytic processing resulting in 13 amino-acid long mature peptides with hydroxylated prolines in the fourth and the seventh positions (Okamoto et al., 2013). In addition, the hydroxyproline in the seventh position is glycosylated with three arabinosyl residues (Okamoto et al., 2013) by the activity of the RDN1 and RDN1-related proteins (Schnabel et al., 2011; Kassaw et al., 2017). The glycosylation by the tri-arabinosyl oligosaccharide is absolutely required for the activity of the peptides because mono-arabinosylated peptides or peptides only with hydroxy-prolines have no biological activity (Imin et al., 2018). The hypothesis that a CLE peptide is signal Q that travels from the root to the shoot to bind to HAR1 has been proven by showing that the triple-arabinosylated peptide CLE-RS2 is transported through the xylem of *L. japonicus* and binds directly to HAR1 (Okamoto et al., 2013). On the other hand, the nature of the SDI, which is produced after CLE-mediated activation of its shoot receptor is less clearly defined but, in *L. japonicus*, it involves cytokinin production in the shoot *via* the CLE-HAR1/SUNN/NARK-activated isopentenyltransferase gene *IPT3*, suggesting that SDI is a cytokinin derivative or that the shoot-derived cytokinins generate a secondary signal (Sasaki et al., 2014). In line with this hypothesis, it was shown by petiole feeding of soybean leaf extracts from AON-induced plants that SDI is a small molecular weight (<1 kDa) molecule that is heat-stable and resistant against the activity of proteases and RNases (Lin et al., 2010). The inhibition of nodulation in the roots by SDI further requires the F-box protein Too Much Love (TML), whose target molecules are not yet known (Takahara et al., 2013; Sasaki et al., 2014).

### CEP Peptides, Positive Effectors of Nodulation Efficiency

Another class of post-translationally modified peptides, the CEP molecules, was also found to regulate systemically nodule formation but unlike the CLE peptides, having a positive effect on nodule number. These peptides are involved in controlling other developmental processes as well, such as lateral root development and nitrate transporter deployment. All these functions are related to assuring the adequate nitrogen supply for plants, and by this means, CEPs can be central molecules coordinating these processes (Taleski et al., 2018). Several *CEP* genes were induced by low-nitrogen conditions in *M. truncatula* (Imin et al., 2013). Among them, *MtCEP1* was shown to positively influence the number of nodules on *M. truncatula* roots and at the same time to negatively control lateral root formation. Both overexpression of *MtCEP1* and adding synthetic MtCEP1 peptide resulted in increased nodule number and size, as well as more efficient nitrogen-fixation, and even partially tolerating high-nitrogen levels, which typically strongly suppresses nodulation (Imin et al., 2013). MtCEP1 peptide treatment also increased the root competency for nodule development as well as infection thread formation. MtCEP1 could also alleviate the inhibitory effects of increased ethylene-precursor levels on nodulation without affecting the ethylene production (Mohd-Radzman et al., 2016). This work has revealed an interface between MtCEP1 and the phytohormone-mediated signaling that regulates nodulation efficiency and plant susceptibility to infection in *M. truncatula*. Genetic evidence presented by Mohd-Radzman et al. (2016) also proved that the positive effect of *MtCEP1* on nodulation is dependent on *COMPACT ROOT ARCHITECTURE 2 (CRA2*) (Huault et al., 2014) through—at least in part—the ethylene signal-transduction pathway including MtEIN2/SKL (ETHYLENE INSENSITIVE2; SICKLE) (Penmetsa and Cook, 1997; Oldroyd and Downie, 2008). CRA2, which is the putative receptor of MtCEP1, was shown to act positively on root nodule formation systemically from the shoot (Huault et al., 2014), however, independently from the AON regulation. Thus, CRA2 and MtCEP1 represent a new systemic circuit of regulation on nodulation.

The functional, 15 amino acid long CEP family members are processed from non-functional prepropeptides and decorated with similar post-translational modifications as CLE peptides. *CEP* genes encode prepropeptides with an N-terminal secretion signal sequence, a variable domain, one or more conserved CEP domains, and one or more flanking variable regions (Ogilvie et al., 2014). MtCEP1 has two conserved CEP domains, D1 and D2. CEPs are frequently hydroxylated at various proline residues and the pattern of hydroxylation has an influence on their biological activity (Delay et al., 2013; Imin et al., 2013; Mohd-Radzman et al., 2015). MtCEP1 D1 peptide variants were also identified with tri-arabinosylation at proline in the 11th position (Mohd-Radzman et al., 2015). A recent work by Patel et al. (2018) analyzed the secreted peptidome of *Medicago* hairy root cultures and xylem sap and found completely new versions of the MtCEP peptides. Some of them possessed unexpected N- and C-terminal extensions that suggested roles for endo- and exoproteases in CEP peptide maturation. These authors determined not only the structure of these molecules with various length and modifications but also chemically synthesized different MtCEP1 D1 variants to test their biological activities. The peptides with N-terminal extensions were unable to increase root nodule number, while the variant with only one amino acid C-terminal extension had biological activity. Unexpectedly, tri-arabinosylated MtCEP1 D1 derivatives had a reduced capacity to increase nodule numbers. Thus, this post-translational modification seems to have a different effect on the biological activity of CLE and CEP peptides. It remains to be determined whether these modifications affect the perception of the CEPs by their receptor and what are the elements involved in the transduction of the CEP signal. Furthermore, the exact biological meaning of tri-arabinosylation of the CEP peptides needs further analysis. The intriguing opposing effect of this post-translational modification on nodule-inhibiting CLE and nodule-stimulating CEP suggests that arabinosylation of peptides plays key regulatory roles in the peptides’ activity controlling nodule numbers that can integrate the AON and CRA2/CEP regulatory circuits.

## Nodule-Specific Peptides Targeted to the Symbiosome

### Nodule-Specific Peptides Governing Terminal Bacteroid Differentiation

Beijerinck (1888) described the bacteroids in *Vicia faba* nodules as “derived from bacteria by a metamorphic process, that have lost their ability to reproduce… They are derived from normal *Bacillus radicicola* (probably a *Rhizobium leguminosarum* sp.) by a stepwise loss in their power of reproduction. Bacteria that are still capable of growth on gelatin plates can be isolated in large numbers from the very young root nodules, as well as from the actively growing regions of older root nodules.” This remarkably precise account, made 130 years ago, is one of the first descriptions of bacteria housed in legume nodules and it drew already the attention to the striking differentiation process of the bacteria in nodules. Since then, this process has been on and off (but mostly off) the scientific agenda of researchers in the field, and it is only since the last 15 years, with the discovery of the NCR peptides that we see a renewed interest. Beijerinck made drawings of large, often *Y*-shaped terminally differentiated bacteroids. Such bacteroids were observed in the nodules of *Vicia, Pisum*, and *Medicago* species belonging to the IRLC and were thought to be characteristic features of the indeterminate nodules. Later works revealed that (i) terminal bacteroid differentiation is not universal in the legume family and depends on the genetic repertoire of the host plant (Mergaert et al., 2006); (ii) it is not a general characteristic of the indeterminate nodules (Ishihara et al., 2011); (iii) the ability to direct bacterial differentiation into swollen (most probably terminally differentiated) bacteroids evolved independently in five out of the six investigated subclasses of the Papilionoideae subfamily (Oono et al., 2010); (iv) terminally differentiated bacteroids fix nitrogen more efficiently than unaltered ones (Sen and Weaver, 1984; Oono and Denison, 2010); (v) that the process is in large part determined by nodule-specific peptides called NCRs and possibly other secreted peptides. Comparative nodule transcriptome analysis of two model legumes, *M. truncatula* and *L. japonicus* hosting terminally differentiated and unaltered bacteroids, respectively, in their nodules identified three gene families in *M. truncatula* encoding secreted peptides that are missing from the *L. japonicus* transcriptome (Kevei et al., 2002; Mergaert et al., 2003; Laporte et al., 2010; Trujillo et al., 2014). These families, called the NCRs, the GRPs, and the SNARPs are described below.

### The Nodule-Specific NCRs of *M. truncatula*

#### The Extremely Large *NCR* Gene Family

The genes in the largest family with over 700 members in *M. truncatula* code for peptides termed nodule-specific cysteine-rich (NCR) peptides (Mergaert et al., 2003). Nearly all *NCR* genes are exclusively expressed in the infected cells of the nodules (Mergaert et al., 2003; Guefrachi et al., 2014). The gene products are characterized by four or six cysteines in conserved positions in the otherwise extremely divergent mature peptide sequence and by a relatively conserved signal peptide sequence. The structure of the NCR peptides resembles that of defensins, innate immunity effectors in plants, which have the capacity to target and kill infecting microbes. Many NCRs have indeed antimicrobial activity; however, they are different from defensins in many aspects (Maróti et al., 2011; Maróti and Kondorosi, 2014). Unlike defensins, NCR peptides have no role in immunity; they only have a function in symbiosis and are targeted to the bacteroids as was shown by immunological methods (Van de Velde et al., 2010) and by detecting over 200 peptides in the bacteroid proteome (Durgo et al., 2015; Marx et al., 2016). The plethora of NCR peptides, evolving with gene duplication and diversifying selection, reflects likely multiple interactions with bacterial targets and many diverse modes of actions.

#### NCR Peptides Control Terminal Bacteroid Differentiation

Treating rhizobia with NCR peptides or ectopic expression of NCR genes in legumes devoid of NCRs provoked symptoms of terminal differentiation (endoreduplication of the bacterial genome together with enlargement of the cell, loss of cell division capacity, increased membrane permeability) indicating that these peptides govern the differentiation process (Van de Velde et al., 2010). Further evidence came by blocking the transport of NCR peptides to the bacteroids in the *M. truncatula* signal peptidase complex mutant, which resulted in the complete absence of bacteroid differentiation (Van de Velde et al., 2010; Wang et al., 2010). The mode of action of the more than 600 NCR peptides in *M. truncatula* remains elusive except for a few cases and their activity might be quite different based on the low sequence similarity of the individual members. The high diversity in amino acid sequence and composition of the mature peptides provide large variations in their physicochemical properties, which is reflected also by the wide spectrum of isoelectric points (p*I*) ranging from 3.5 to 10.5. Roughly one-third of the NCRs are cationic while the rest are anionic or neutral (Montiel et al., 2017).

Most studies have been focused on the cationic NCR peptides as *in vitro* they possess strong antimicrobial activity against a wide range of Gram-negative and Gram-positive bacteria as well as unicellular and filamentous fungi (Tiricz et al., 2013; Ördögh et al., 2014). This bactericidal and mycocidal activity is mediated *via* the disruption of the integrity of microbial membranes (Ördögh et al., 2014; Mikuláss et al., 2016). However, these experiments were performed at high concentrations of synthetic NCR peptides, which do not reflect and are most likely incomparable with the peptide concentrations in the nodule cells where, in addition, many other NCRs, cationic and non-cationic ones are present that might act together.

Terminal bacteroid differentiation is accompanied by endoreduplication when the genome of the bacteria duplicated without cell division. A crucial step of the cell division is the formation of the *Z*-ring, assembled by polymerization and localization of the FtsZ protein at the future site of the septum, required for separating the mother and daughter cells (Lutkenhaus and Addinall, 1997). The cationic NCR247 peptide does not provoke membrane damage at sub-lethal concentrations but enters the bacterial cytosol and drastically alters the physiology of the bacterium manifested by disappearance of proliferating cells and appearance of septum-less elongated cells. The NCR247 peptide binds to FtsZ and this interaction abolishes polymerization of FtsZ and thereby septum formation (Farkas et al., 2014). Interestingly, the same symbiotic cells produce another peptide, NCR035, which in growing bacterial cultures localizes to the septum and this localization can be abolished by treating rhizobia with the NCR247 peptide (Farkas et al., 2014). Thus, it seems that more than one peptide might affect single biological pathways and processes, particularly those with key importance in symbiosis, such as stopping bacterial proliferation in the host cell. Moreover, the NCR247 peptide attenuates the expression of critical cell cycle regulator genes *ctrA, gcrA, dnaA* as well as cell division genes, including genes required for *Z*-ring function, among others (Tiricz et al., 2013; Penterman et al., 2014), another way to regulate the cell cycle of the developing bacteroids. In addition, this peptide inhibits translation not only by downregulating the expression of ribosomal genes but also *via* binding to several ribosomal proteins. NCR247 might thus contribute to the altered proteome and physiology of the bacteroids. These effects could be amplified by binding of NCR247 to the GroEL chaperon modifying presumably interaction of GroEL with other proteins (Farkas et al., 2014).

#### Other Roles of NCR Peptides

At present, it is unknown what the role and the extent of the NCRs’ antimicrobial activity are during symbiosis. In addition to the expected much lower NCR concentrations in the nodules than the ones used in *in vitro* experiments, non-cationic peptides present in the NCR cocktails produced by a given symbiotic cell might counteract the killing effect of the cationic peptides. An intriguing possibility is that the cationic peptides facilitate somehow the uptake of the acidic and neutral ones or act in complexes in the membranes and/or in the cytoplasm. Indeed, NCR247 was shown to interact with two anionic NCRs (Farkas et al., 2014), but the promotion of uptake of these peptides by NCR247 has not been demonstrated. The antibacterial activity of NCRs likely keeps rhizobia on the verge of destruction, manifested in the immediate and NCR-dependent death of *bacA* mutant bacteria in the nodule. The BacA protein is a peptide transporter that provides tolerance toward the antimicrobial activity of the NCR peptides (Haag et al., 2011).

The role of neutral and anionic NCRs, on the other hand, is a great enigma. They were shown to accumulate in the bacteroids and they might be major players providing a plethora of novel activities. However, at present, it is unknown how they enter the bacteria and what they do there. Unlike the cationic NCRs, none of the tested neutral or anionic NCRs showed antimicrobial activity (Tiricz et al., 2013; Ördögh et al., 2014). The NCR211 required for the development of effective nodules (see below) is so far the only anionic peptide, which has a mild antibacterial activity (Kim et al., 2015).

Despite the high amino acid sequence variation, the large number of NCR peptides suggested redundant functions making their genetic analysis difficult. However, map-based cloning from plant mutants unable to establish nitrogen-fixing symbiosis led to the identification of single peptides (NCR169 and NCR211) that are required for the development of the effective interaction. Their absence resulted in the arrest of bacteroid differentiation and/or in the loss of bacteroid persistence (Horváth et al., 2015; Kim et al., 2015) *via* a mechanism unknown at present. An interesting and kind of opposing activity of the NCR peptides is their involvement in the selection of the bacterial partner: Incompatibility between *M. truncatula* ecotype Jemalong and *Sinorhizobium meliloti* strains Rm41 and A145, which form effective symbiosis with other *Medicago* partners, results in the elimination of the bacterial partner from the nodule region where nitrogen fixation should take place. This elimination is mediated by allelic variants of two NCR peptides, called NFS1 and NFS2, but the process cannot be explained by a stronger antimicrobial activity of the incompatible variants because the sensitivity of compatible and incompatible bacteria is quite similar (Yang et al., 2017; Wang et al., 2017, 2018). The phenotypes of mutants in *NCR169* and *NCR211* and the *NFS1* and *NFS2* alleles clearly suggest that antimicrobial activity is not the only mode of action of members of the NCR family.

#### The Evolution of NCRs in the IRLC

The IRLC is a clade of legumes, which is mostly constituted by temperate herbaceous tribes such as the *Galegeae, Carmichaelieae, Cicereae, Hedysareae, Trifolieae, Vicieae*, as well as the tropical tribe Millettieae (*Callerya, Wisteria*, and related genera) (Wojciechowski et al., 2000). Based on similarity searches, NCRs were first recognized in nodule EST sequencing data of several IRLC species from distinct genera and subclades (Frühling et al., 2000; Györgyey et al., 2000; Jimenez-Zurdo et al., 2000; Crockard et al., 2002; Fedorova et al., 2002; Kaijalainen et al., 2002; Kato et al., 2002; Mergaert et al., 2003; Chou et al., 2006). Nodule transcriptome sequencing from species representing the main subclades (Hedysaroid, Astragalean, and Vicioid) of the IRLC as well as analysis of RNA-Seq data from other IRLC species shed light on the evolution of NCRs in the IRLC (Montiel et al., 2017). It was shown that the numbers of NCR genes are highly variable (from 7 to >700) and expanded independently in different lineages of IRLC legumes. In nodules of *Glycyrrhiza uralensis* (the most basal IRLC legume) infected with *Mesorhizobium tianshanense*, only seven NCR genes have been identified, none of them encoding peptides with positive charge (Montiel et al., 2017). *M. tianshanense* bacteroids display symptoms of terminal differentiation, however, the swelling of the bacteroids represents a mild morphological response, compared to the drastic enlargement of *Y*-shaped bacteroids in several Vicioid legumes (Montiel et al., 2016). The small cocktail of NCRs produced by *G. uralensis* and the absence of cationic peptides seems to be insufficient to induce irreversible differentiation in *Sinorhizobium fredii*, a rhizobial strain abnormally resistant to the antimicrobial action of NCR247 and NCR335 (Crespo-Rivas et al., 2016). The GuNCRs identified until now, are likely the ancestor symbiotic peptides in the IRLC, since each of them have at least one putative ortholog in another IRLC legume from different genera (Montiel et al., 2017). The presence of recognizable orthologs between different genera is, however, rather rare. For example, only a few orthologs can be predicted among the closely related species *M. truncatula* and *M. sativa*. The Vicioid legume *Cicer arietinum* represents a peculiar case, where 20 CaNCRs have putative orthologs in the non-Vicioid species *G. uralensis, O. lamberti, A. canadensis*, and *O. viciifolia*, but surprisingly none in any Vicioid legume. In addition, the 63 NCRs found in the nodule transcriptome of *C. arietinum* represent a considerably low number, compared to the large gene families in *Galega orientalis, Ononis spinosa, Pisum sativum*, and *Medicago* spp., all of them part of the Vicioid subclade (Wojciechowski et al., 2004; Montiel et al., 2017). Additionally, the swollen and spherical bacteroids of *C. arietinum* nodules contrast with the elongated-branched bacteroids in other legumes located in the same subclade such as *Medicago* sp., *G. orientalis, P. sativum, V. faba*, and *Trifolium repens* (Mergaert et al., 2003, 2006; Montiel et al., 2016). In general, a positive correlation was found between the degree of bacteroid elongation and the number of the expressed *NCR*s (Montiel et al., 2017). Legumes with elongated-branched bacteroids express hundreds of NCRs, characterized by a large proportion of cationic peptides with a well-defined isoelectric point (Montiel et al., 2017). Spherical bacteroids can be found both in *C. arietinum* and *O. spinosa* nodules; however, these species share no evident NCR pattern (Lee and Copeland, 1994; Montiel et al., 2016; Montiel et al., 2017). These clues indicate that *NCR* gene families took different evolutionary trajectories, showing variable duplication rates (Alunni et al., 2007; Montiel et al., 2017) that were likely favored by transposable elements located in flanking regions of *NCR* genes as shown in *M. truncatula* (Satgé et al., 2016). Clearly, the enrichment of cationic NCRs with particular isoelectric points had great impact on the morphology of the hosted endosymbionts.

#### NCR Genes in Dalbergoid Legumes

Terminal bacteroid differentiation is not restricted to the IRLC legumes (Oono et al., 2010). The Dalbergoid clade is one of the other legume groups in which bacteroids, similar to the IRLC legumes, differentiate into polyploid and strongly enlarged bacteria (Czernic et al., 2015). Depending on the host species, these bacteroids have either an elongated morphology similar as in *Medicago* (e.g., *Aeschynomene afraspera, Aeschynomene nilotica*) or they can be almost perfect, large spheres as in *C. arietinum* and *O. spinosa* (e.g., in *Aeschynomene indica, Aeschynomene evenia*, and *Arachis hypogaea*). Transcriptome analysis in different *Aeschynomene* species identified a family of peptide genes with similar features as the IRLC NCRs. They are secretory peptides, characterized by conserved cysteine motifs in the mature domain. However, the *Aeschynomene* NCR peptides have no sequence similarity to the IRLC NCRs [for example, the spacing and number (six or eight) of cysteines is different]. They form thus a separate family of peptides. The genes encoding the *Aeschynomene* NCRs are only expressed in nodules and they are activated just before the onset of bacteroid differentiation. They are expressed only in the symbiotic nodule cells and a proteome analysis of purified bacteroids demonstrated that the peptides are targeted to them. Moreover, blocking the secretory pathway by RNAi targeting one of the subunits of the signal peptidase complex inhibits bacteroid differentiation (Czernic et al., 2015) as was described before in *M. truncatula* mutated in the orthologous gene (Van de Velde et al., 2010). Another parallel with the bacteroid differentiation in *Medicago* is the requirement of a BacA-like peptide transporter, named BclA, in *Bradyrhizobium* symbionts for the interaction with *Aeschynomene* (Guefrachi et al., 2015). In the absence of this transporter in the *bclA* mutant, the bacteroids do not differentiate into their polyploid and elongated forms and die, exactly as the phenotype of the *S. meliloti bacA* mutant in *Medicago* nodules (Haag et al., 2011).

Together, these similitudes between the *Aeschynomene* and the IRLC suggest that bacteroid differentiation in the Dalbergioid clade, which evolved independently from the bacteroid differentiation in the IRLC clade (Oono et al., 2010), is based on very similar mechanisms used by IRLC legumes. Nevertheless, some unresolved questions remain. One of them is that a recent transcriptome analysis in *A. hypogaea* failed to detect homologs of the *Aeschynomene NCR* genes (Karmakar et al., 2018). However, this study identified another, small, family of cysteine-containing peptides, related to the antimicrobial PR-1 family and the authors suggested the involvement of these peptides in bacteroid differentiation. Alternatively, the *A. hypogaea* and *Aeschynomene* NCR peptides diverged too much to be identified by homology and a specific bioinformatics search for small, secreted, and cysteine-rich peptides would be required to identify them. A second open question that requires further investigation is the absence of detectable *in vitro* activity of the *Aeschynomene* peptides (Czernic et al., 2015). As described above, many of the *Medicago* peptides show a strong action against bacteria, including arrest of division and membrane permeabilization and complete cell lysis (Tiricz et al., 2013; Ördögh et al., 2014; Mikuláss et al., 2016). None of the thus far tested *Aeschynomene* peptides displayed such an activity on the *Brabyrhizobium* symbionts of *Aeschynomene* nor on any other tested bacterium including ones that show a strong response to the *Medicago* peptides. All identified peptides in the *Aeschynomene* transcriptome are either neutral or anionic while the most active antimicrobial NCR peptides of *Medicago* are positively charged. In addition, the *Bradyrhizobium* symbionts seem to be very robust bacteria that are highly resistant to antimicrobial peptides including the most active *Medicago* NCR peptides with a broad spectrum of activity. This robustness of bradyrhizobia is due to their very tough cell envelope. Contrary to most other rhizobia, bradyrhizobial envelopes contain hopanoids, a class of bacterial lipids, similar to eukaryotic steroids (cholesterol). They are known to render bacterial membranes more rigid and resistant to membrane stresses, including ones caused by antimicrobial peptides (Belin et al., 2018). And indeed, hopanoid mutants of *Bradyrhizobium* become more sensitive to *Medicago* NCR peptides and other antimicrobial peptides (Kulkarni et al., 2015). Altogether, this leaves open the question how the *Bradyrhizobium* symbionts in the symbiotic cells of *Aeschynomene* nodules are manipulated by the host to respond to these host signals.

The formation of spherical bacteroids in some hosts like the Dalbergoid *A. indica* or *A. evenia* as well as in the IRLC *C. arietinum* and *O. spinosa* is an additional unsettled point. In *A. indica*, the spherical bacteroids are formed through an intermediate stage of elongation similar to the bacteroids in other *Aeschynomene* or in *Medicago* (Czernic et al., 2015). It is unknown if the transition from elongated to spherical morphotypes is the result of the action of specific NCR peptides or of another host factor.

#### Nodule-Specific Glycine-Rich Proteins (GRPs)

Glycine-rich protein-encoding genes are a second group of nodule-specific transcripts that seems to be restricted to the IRLC. They were originally identified in *V. faba* and *Medicago* spp. (Küster et al., 1995; Schröder et al., 1997; Györgyey et al., 2000; Jimenez-Zurdo et al., 2000; Kevei et al., 2002; Alunni et al., 2007). The *GRP* gene family is much smaller than the NCR one with less than 30 members in *M. truncatula* (Alunni et al., 2007).

Glycine-rich proteins have been described in a wide variety of plant species performing variable roles including activity in biotic and abiotic interactions of the plants with their environment (Sachetto-Martins et al., 2000). Semi-repetitive glycine regions characterize GRP sequences that can be classified according to the presence of different binding motifs or a signal peptide (Mangeon et al., 2010). Usually, GRPs have around 80% glycine content arranged in specific motifs, but the nodule expressed secreted GRPs are shorter polypeptides than the usual GRPs and possess only 20–30% glycine residues without any recognizable motif. Interestingly, the signal peptide sequence of the nodule-specific GRPs found in IRLC legumes is also a distinctive feature not shared with the signal peptides of GRPs from other plant species (Kevei et al., 2002; Alunni et al., 2007).

A recent search for GRPs revealed that these peptides are also expressed in the nodules of representative species from the Astragalean and Hedysaroid subclades along with *G. uralensis* (J. Montiel, unpublished). However, the size of GRP families is considerably lower in the non-Vicioid species compared to the Vicioid legumes *G. orientalis, C. arietinum, O. spinosa*, and *Medicago* spp. Unlike the *NCR* gene families, the enrichment and diversification of the *GRP* families show no correlation with the morphotype of the hosted bacteroids, and rather seems to be specific for members of the Vicioid subclade (J. Montiel, unpublished). The expression profile of the *GRP*s in the different nodule zones is another relevant difference to *NCR*s. In *M. truncatula*, 39% of *GRP* transcripts are present in the infection zone, the nodule tissue where bacteroid differentiation takes places (J. Montiel, unpublished), while this region contributes only to 18% of *NCR* transcripts (Roux et al., 2014; Montiel et al., 2017). This observation indicates that several GRPs are potentially involved in bacteroid differentiation (Kevei et al., 2002; Kondorosi et al., 2013). Gene characterization of different *GRP*s through reverse genetics could help to understand the role(s) played by these proteins in nodulation and their high diversification within Vicioid legumes.

#### SNARPs or LEED..PEEDs

The SNARPs or LEED..PEEDs form a small family, 10–13 members, of small secreted and nodule specific peptides in *Medicago* (Laporte et al., 2010; Trujillo et al., 2014). These peptides are not longer than 70 amino acids and are characterized by one or two conserved domains of acidic amino acid residues, the LEED or PEED domains, hence, one of their names (Trujillo et al., 2014). They were also characterized as RNA-binding peptides, from there, the other name, small nodulin acidic RNA-binding protein, or SNARP in short (Laporte et al., 2010). Intriguingly, this peptide family is specific to the *Medicago* lineage (*M. truncatula* and *M. sativa*) because homologous sequences are absent in all other genomes of legumes or other plant species, showing that the family arose within this clade during the past 25 million years (Trujillo et al., 2014). Their expression, similarly to the above described NCR and GRP peptides, is absolutely nodule specific with transcripts only found in the distal and proximal infection zones, the interzone and the nitrogen fixation zone of nodules while they are absent in all other plant tissues. This expression pattern suggests a specific role of these peptides in the later stages of nodule development, potentially in symbiotic cell differentiation or bacteroid formation. As described above, two members of the family, SNARP1 and SNARP2 were identified in a yeast three-hybrid screen for proteins that interact with the *MtENOD40* mRNA (Campalans et al., 2004). *In vitro* biochemical studies further demonstrated that the SNARP2 protein has non-specific binding activity to single-stranded RNA (Laporte et al., 2010). On the other hand, the LEED..PEED/SNARP proteins are secretory proteins indicating that they should be localized in the endomembrane system, the symbiosomes, or in the extracellular space (Laporte et al., 2010). How these putative localizations, which—except for the bacteroids in the symbiosomes—supposedly do not contain RNA, can be reconciled with the RNA-binding activity of these peptides needs further investigation. However, even if their molecular role is still unclear, the importance of SNARP peptides in nodule development is strongly supported by RNAi inactivation of the *MtSNARP2* gene, which led to the formation of abnormal nodules (Laporte et al., 2010). In these nodules, infection of symbiotic cells and bacteroid formation seemed to proceed normally but the symbiotic cells and their bacteroids were not stably maintained and degenerated prematurely. Thus, even if these reverse genetic experiments conclusively demonstrate the importance of the SNARP peptides for normal symbiotic cell formation, they raise at the same time new questions. Why are these peptides essential in *Medicago* nodules while these peptides are absent in closely related legumes such as pea, clover, or chickpea (Trujillo et al., 2014), which form nodules very similar in structure and function to the *Medicago* nodules?

## Concluding Remarks

The large number of different peptides and peptide families that we have described here are those for which at least a minimal amount of evidence demonstrates a specific role in symbiosis. But they might just as well be only the tip of the proverbial “peptide-iceberg.” Small proteins have traditionally escaped gene prediction efforts in plant genomes because algorithms were biased against them by a concern to avoid wrongful annotations. However, in recent years, predicting peptide genes in plant genomes by dedicated bioinformatics tools have provided the insight that functional genes encoding small peptides are massively hidden in plant genomes (Silverstein et al., 2005, 2007; Lease and Walker, 2006; Hanada et al., 2013; Pan et al., 2013; Ghorbani et al., 2015; de Bang et al., 2017). Combined with transcriptomics, peptide predictions have been the impetus for the functional characterization of many of the above described peptides. In a recent large scale effort, the Mt4.0 and Mt3.5v5 releases of the *M. truncatula* genome were re-annotated using a suite of bioinformatics programs with the specific aim to search for ORFs encoding small secreted peptides (SSPs) (de Bang et al., 2017). This approach yielded a comprehensive catalog of almost 2,000 genes from 46 previously defined SSP families, including all the above described families. In addition, another catalog of almost 2,500 genes encoding putative novel SSPs was established. Focusing on SSP genes, known or suspected to function *via* receptor-mediated signaling, a transcriptome analysis by RNA-seq was performed during a time course of nodule formation and in response to Nod factors. This analysis revealed 365 differentially expressed known signaling SSPs plus an additional several hundred genes encoding putative novel SSPs. The very large majority of these differentially expressed genes were up- or downregulated in developing or mature nodules. Their differential regulation during the early stages of nodulation and Nod factor signaling has not yet been tested thoroughly. But in any case, these results suggest an unanticipated complexity and importance of peptide-mediated signaling in the orchestration of the symbiosis.

## Author Contributions

AK, PM, JM, GE, and ÉK wrote the manuscript and read and approved the final version of the manuscript.

## Conflict of Interest Statement

The authors declare that the research was conducted in the absence of any commercial or financial relationships that could be construed as a potential conflict of interest.
